# Curcumin-Mediated Resistance to Lenvatinib via EGFR Signaling Pathway in Hepatocellular Carcinoma

**DOI:** 10.3390/cells12040612

**Published:** 2023-02-14

**Authors:** Katsuki Miyazaki, Yuji Morine, Caiming Xu, Chiharu Nakasu, Yuma Wada, Hiroki Teraoku, Shinichiro Yamada, Yu Saito, Tetsuya Ikemoto, Mitsuo Shimada, Ajay Goel

**Affiliations:** 1 Department of Molecular Diagnostics and Experimental Therapeutics, Beckman Research Institute of City of Hope Comprehensive Cancer Center, Duarte, CA 91010, USA; 2 Department of Surgery, Tokushima University, Tokushima 779-1510, Japan

**Keywords:** Lenvatinib, Curcumin, EGFR, hepatocellular carcinoma, PI3K-AKT, resistance

## Abstract

Lenvatinib is a multi-kinase inhibitor approved as a first-line treatment for patients with unresectable advanced hepatocellular carcinoma (HCC). However, its response rate is unsatisfactory, primarily due to the acquisition of resistance, which limits its clinical significance for treating patients with HCC. Recent evidence suggests that epidermal growth factor receptor (EGFR) activation can trigger Lenvatinib-resistance; and is considered an important therapeutic target in HCC. Curcumin, one of the most studied naturally occurring botanicals with robust anti-cancer activity, is also reported to be a potent tyrosine kinase inhibitor. In this study, we hypothesized that the anti-EGFR potential of Curcumin might help overcome Lenvatinib resistance in HCC. We established two Lenvatinib-resistant cells and discovered that a combination of Curcumin and Lenvatinib exhibited a synergistic anti-tumor efficacy in the resistant HCC cell lines. In line with previous reports, Lenvatinib-resistant cell lines revealed significant activation of the EGFR, and genomewide transcriptomic profiling analysis identified that the PI3K-AKT pathway was associated with Lenvatinib resistance. The combination treatment with Curcumin and Lenvatinib dramatically suppressed gene and protein expression of the EGFR-PI3K-AKT pathway, suggesting Curcumin overcomes Lenvatinib resistance via inhibition of EGFR. We further validated these findings in tumor spheroids derived from resistant cell lines. In conclusion, we, for the first time, report that Curcumin reverses Lenvatinib resistance in HCC, and that their combination has clinical application potential for adjunctive treatment in HCC.

## 1. Introduction

Hepatocellular carcinoma (HCC) is the most common primary liver malignancy and the third leading cause of cancer-related deaths [1]. Patients with advanced, unresectable HCC are often treated with systemic therapies, and currently, Sorafenib [2], Lenvatinib [3], and Atezolizumab plus Bevacizumab [4] are approved as first-line treatments. Among these first-line therapies in unresectable HCC, Atezolizumab and Bevacizumab are the first choices [5,6]. However, growing evidence indicates that immune therapies might be less effective in HCC patients with a non-viral etiology, which is currently on the rise worldwide [7,8]. More recently, a large international propensity score matching analysis for the first time revealed that Lenvatinib is associated with a significant survival benefit compared to Atezolizumab plus Bevacizumab among patients with non-viral and nonalcoholic steatohepatitis related-HCC [9,10]. Moreover, in terms of cost-effectiveness, several studies have shown that Atezolizumab plus Bevacizumab was not a superior option to Sorafenib or Lenvatinib [11,12,13,14]. In addition, Lenvatinib has received increasing attention as a combination treatment with transcatheter arterial chemoembolization (TACE) [15,16,17]. In the prospective phase III, LAUNCH trial, comparing Lenvatinib monotherapy and Lenvatinib plus TACE sequential therapy (LEN-TACE), data indicated a significantly longer overall survival and progression-free survival in the LEN-TACE group vs. those who received Lenvatinib monotherapy [18]. These studies highlight the clinical importance of Lenvatinib in patients with HCC, even in the era of immunotherapy.

However, the overall response rate of Lenvatinib is only ~24% [19], and even among responder patients, the subsequent acquisition of resistance limits its therapeutic efficacy. Nonetheless, an objective response to Lenvatinib is an independent prognostic factor among patients with an unresectable HCC [20]. Although the triggers of Lenvatinib resistance remain unclear, recent studies have shown that epidermal growth factor receptor (EGFR) activation is a frequent phenomenon in Lenvatinib-resistant cells [21,22], and a combination of Lenvatinib and Gefitinib, an anti-EGFR drug, offers promising survival benefits in early phase clinical trials [23]. Hence, while the combined treatment approaches using Lenvatinib and EGFR inhibitors have clinical potential, the significant challenges, including their toxicity and high costs, present a challenge for their routine use in clinical settings.

Several naturally occurring botanical compounds have recently gained more attention from researchers and clinicians because of their inherent multi-targeted and safer therapeutic efficacy compared to conventional chemotherapy and targeted drugs [24,25,26,27,28]. Among these, Curcumin, extracted from Curcuma longa, is one of the most studied compounds due to its potent anti-cancer activity in various malignancies [25,29,30,31]. Curcumin is a chemo-sensitizer against many chemotherapies, but offers simultaneous protective effects on normal organs [30,32]. We have already reported Curcumin’s anti-cancer effects in colorectal and pancreatic cancer [26,32,33]. In line with our previous studies, Curcumin has also been shown to possess anti-tumor activity in HCC [34]. Curcumin-mediated anti-cancer effects are primarily modulated by several important cancer-related processes, such as the inhibition of inflammation, cell cycle arrest of tumor cells, and induction of apoptosis [33,35,36]. Some studies have reported that Curcumin acts as a potent tyrosine kinase inhibitor [36,37]. By virtue of this, Curcumin can target receptor tyrosine kinases, including EGFR, fibroblast growth factor receptor (FGFR), vascular epidermal growth factor receptor (VEGFR), etc. [36]. Lenvatinib is a multi-kinase inhibitor targeting FGFR, VEGFR, platelet-derived growth factor receptor (PDGFR), RET, and KIT [19]. Therefore, we hypothesized that Curcumin could enhance the anti-tumor effects of Lenvatinib as a tyrosine kinase inhibitor, and that its anti-EGFR potential could help overcome the Lenvatinib resistance in HCC. In the current study, we performed a series of systematic studies to reveal that Curcumin reverses Lenvatinib resistance in HCC, and such a combination treatment has potential clinical application in HCC.

## 2. Materials and Methods

### 2.1. Cell Lines and Reagents

Five human HCC cell lines (Huh-7, SNU449, PLC-PRF-5, SNU398, and Sk-Hep-1) were examined in this study. SNU449 was obtained from the American Type Culture Collection (ATCC, Manassas, VA, USA). Huh-7 and PLC-PRF-5 (PLC) were obtained from the RIKEN BioResource Center Cell Bank (Tsukuba, Japan). Dr. Ke, Department of Diabetes Complications and Metabolism, Beckman Research Institute of City of Hope, kindly provided us with SNU398 and Sk-Hep-1 cell lines. All cell lines were cultured in Dulbecco’s modified Eagle’s medium (DMEM; Gibco, Carlsbad, CA, USA) supplemented with 10% fetal bovine serum (Gibco, Waltham, MA, USA) and 1% penicillin-streptomycin. All cells were maintained in a 37 °C incubator with a 5% humidified CO_2_ atmosphere. Curcumin was generously provided by EuroPharma USA, Green Bay, WI, USA, and Lenvatinib was purchased from Sigma-Aldrich, St. Louis, MO, USA.

### 2.2. Cell Viability Assays

For these assays, five thousand cells were seeded into 96-well plates. After overnight incubation for cell adherence to the plates, all cell lines were treated with Lenvatinib, Curcumin, or their combination using different concentrations for 48 h. Following completion of the treatments, MTT assays were performed using the Cell Counting Kit-8 (CCK-8; Dojindo Molecular Technologies, Inc., Kumamoto, Japan) according to the manufacturer’s instructions. The absorbance of the final colored product was analyzed at 450 nm using a plate analyzer (Molecular Devices, San Jose, CA, USA). To evaluate the effects of the drug combination, isobolograms were generated using ComboSyn software (ComboSyn, Inc., Paramus, NJ, USA), and the combination index (CI) < 1.0 was considered a synergistic effect.

### 2.3. Development of Lenvatinib-Resistant Cell Lines

Lenvatinib-resistant cells were developed from Huh-7 and PLC cell lines. Parental cells were continuously exposed to a Lenvatinib-supplemented medium. Surviving cell clones were transferred to a medium with a higher concentration of Lenvatinib, and the concentration of the drug was increased by 0.5 μM weekly for 10 months. Finally, resistant cells could stably grow in a 20 μM Lenvatinib-supplemented medium and were selected for final experimentation.

### 2.4. Invasion Assays

All HCC cell lines were seeded in 6-well plates and treated with drugs for 48 h. After treatment, 2.5 × 10^4^ cells were seeded into the Matrigel invasion chambers (Corning, Tehama County, CA, USA) with 8.0 µm pore-sized PET membranes. After another two days of the invasion, invaded cells were fixed and stained by Diff-Quick and counted.

### 2.5. Colony Formation Assays

One thousand cells were seeded in 6-well plates. After 3–5 days of incubation, cells were treated with individual drugs or their combination for 48 h. Following the completion of the treatment period, cells were cultured in a standard culture medium for another week and harvested. The cells were fixed in the culture plates with 100% methanol and stained with 1% crystal violet. Colony formation rates were quantified as the percentages of the stained area by ImageJ 1.53 software (National Institutes of Health, Bethesda, MD, USA), as previously reported [38].

### 2.6. Apoptosis and Oxidase Stress Assay

Apoptosis and oxidative stress assays were performed following the treatment of cancer cell lines with each compound for 16 h. Cell apoptosis and oxidase stress levels were examined by the Muse Cell Analyzer (Millipore Corp, Billerica, MA, USA) using the Annexin V and Dead Cell Kit and the Oxidative Stress Kit, per the manufacturer’s instructions.

### 2.7. Spheroid Formation Assays

The method for these assays was previously described [27,39]. Briefly, Lenvatinib-resistant cell lines were seeded into ultra-low attachment plates in serum-free DMEM-F12 medium (STEMCELL Technologies, Vancouver, BC, Canada) with B27 supplement (Gibco), basic fibroblast growth factor (bFGF; Gibco), and epidermal growth factor (EGF; STEMCELL Technologies). When the small spheroids were formed (after about 2–4 days), they were treated for another 48 h. Following completion of the treatment, the size and number of spheroids were counted using ImageJ 1.53 software.

### 2.8. Identification of Differentially Expressed Genes and Functional Pathway Enrichment in Lenvatinib-Resistant HCC Cells

To discover significantly and differentially expressed genes in Lenvatinib-resistant vs. parental cell lines, we analyzed GSE211850, a publicly available dataset comparing gene expression profiling between the parental and Lenvatinib-resistant Huh-7 cell lines. Significantly upregulated genes were defined if they had a *p* < 0.05 and a Log2FC > 1.0. Subsequently, enrichment analysis of the KEGG pathways was carried out using the DAVID bioinformatic database. The top 50 enrichment pathways were ranked by the number of included genes. In addition, we also examined the prognostic impact of the EGFR pathway in HCC using the GSE10141 dataset.

### 2.9. RNA Extraction and Real-Time Quantitative Reverse Transcription PCR (RT-qPCR) Assays

Total RNA was extracted from cancer cells and spheroids treated with Lenvatinib, Curcumin, or their combination for 16 h. cDNA synthesis was performed using a Reverse Transcription Kit (Thermo Fisher Scientific, Waltham, MA, USA). The qRT-PCR assays were performed using a SensiFAST SYBR Lo-ROX Kit (Bioline, London, United Kingdom) and the QuantStudio 6/7 Flex RT-PCR System (Applied Biosystems, Foster City, CA, USA). GAPDH was used as the housekeeping gene, and gene expression changes were calculated by the delta Ct method. The primer sequences used in this study are described in Table S1.

### 2.10. Western Blotting

Western blotting was performed as previously reported [27,39]. Cells were treated with each compound for 16 h to purify total proteins, which were lysed by RIPA buffer with a proteinase inhibitor cocktail (Thermo Fisher Scientific, Waltham, MA, USA). Purified protein was denatured with 2× Laemmli’s sample buffer (Bio-Rad Laboratories, Hercules, CA, USA), which contained 5% 2-mercaptoethanol (Sigma-Aldrich). The list of antibodies is described in Table S2. The GAPDH protein was used as a loading control, and band intensities were quantified using ImageJ 1.53 software.

### 2.11. Statistical Analysis

All assays were performed in triplicate, and data were described as the mean ± standard deviation. Statistical comparisons were made using the Wilcoxon test (for 2 groups) or the Tukey-Kramer method (for 3 or more groups), and *p* < 0.05 was considered to be statistically significant. The overall survival analysis curves were created using the Kaplan-Meier method, and the differences in patient survival were analyzed by the log-rank test. All analyses were conducted using JMP 8.0.1 (SAS Institute Inc., Tokyo, Japan).

## 3. Results

### 3.1. Curcumin Enhances the Anti-Proliferative Effect of Lenvatinib in HCC Cells

**Figure 1:**
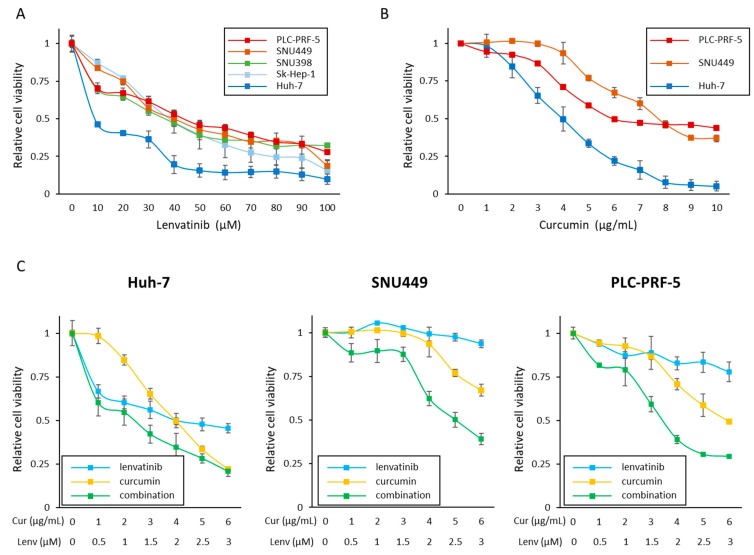
Curcumin enhanced the anti-proliferative effects of Lenvatinib in parental HCC cells. (**A**) Cell viability assay following Lenvatinib treatment for 48 h in five HCC cells. (**B**) Cell viability assay following 48 h of treatment with Curcumin in Huh-7, SNU449, and PLC-PRF-5 cell lines. (**C**) Cell viability assay following Curcumin and Lenvatinib combination treatment for 48 h in Huh-7, SNU449, and PLC-PRF-5 cell lines. Error bars in all panels represent the mean ± SD. SD, standard deviation.

At first, we examined the sensitivity of each HCC cell to Lenvatinib. Cell viability assays following treatment with Lenvatinib (0–100 μM) revealed that Huh-7 cells (the half maximal inhibitory concentration (IC50); <10 μM) were sensitive to Lenvatinib, and SNU398 cells (IC50; 36.6 μM), Sk-Hep-1 (IC50; 38.4 μM), SNU449 (IC50; 40.1 μM), and PLC (IC50; 44.3 μM) were relatively insensitive to Lenvatinib (Figure 1A). To determine the IC50 concentration of Lenvatinib in Huh-7 cells, we performed cell viability assays at lower concentrations of Lenvatinib (0–10 μM), deciphering that the IC50 of Lenvatinib in Huh-7 cells was 2.0 μM (Figure S1). Based upon these findings, we categorized Huh-7 as a Lenvatinib-sensitive cell line, and PLC and SNU449 cell lines as two Lenvatinib-insensitive cell lines for further experiments.

We next evaluated whether Curcumin could exert anti-proliferative effects in these three cell lines (Figure 1B). Curcumin reduced cell viability in all three cell lines in a dose-dependent manner, and the IC50 concentration of Curcumin was 4.0 μg/mL (Huh-7), 7.7 μg/mL (SNU449), and 6.0 μg/mL (PLC) in each of the cell lines. Based on the IC50 concentrations of Lenvatinib and Curcumin in Huh-7 cells (Lenvatinib, 2.0 μM; Curcumin, 4.0 μg/mL), the ratio of the combination treatment was determined to be 1 μM of Lenvatinib: 2.0 μg/mL of Curcumin. We hypothesized that this combined treatment might exhibit a more pronounced antiproliferative effect. In support of our hypothesis, we noted that this combination treatment had the most potent anti-proliferative effects in all three cell lines (Figure 1C). Interestingly, the CI values were < 1.0 (Huh-7; 0.78, SNU449; 0.79, PLC; 0.50) at a concentration of IC50, which indicated the enhanced synergistic antiproliferative effect with the Curcumin and Lenvatinib combination. Moreover, in SNU449 and PLC cell lines, the combination also showed amplified effects at concentrations of IC75 and IC90 (Figure S2). These results suggest that Curcumin can enhance the antiproliferative effects of Lenvatinib in both endogenously Lenvatinib-sensitive and insensitive cell lines.

### 3.2. Successful Establishment of Lenvatinib-Resistant HCC Cell Lines

**Figure 2:**
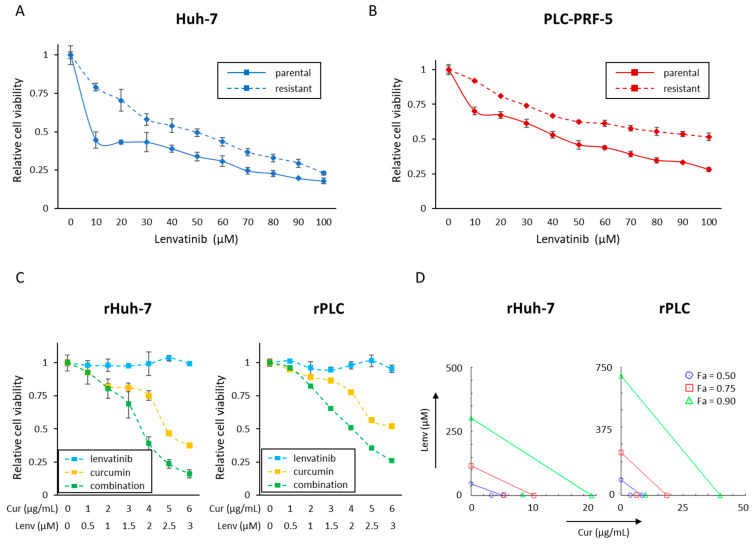
Curcumin reverses the acquired resistance to Lenvatinib in HCC cells. (**A**,**B**) Cell viability assay following Lenvatinib treatment for 48 h in parental and resistant cells of Huh-7 and PLC-PRF-5 cells. (**C**) Cell viability assay following 48 h of Curcumin and Lenvatinib combination treatment in resistant Huh-7 and PLC-PRF-5 cell lines. (**D**) Isobologram analysis of Curcumin and Lenvatinib combination in Huh-7 and PLC-PRF-5 (Lenvatinib-resistant) cell lines. Error bars in all panels represent the mean ± SD. SD, standard deviation.

The primary aim of our study was to determine whether Curcumin could help overcome Lenvatinib resistance in HCC. To achieve this goal, we established two Lenvatinib-resistant cell lines, one from Huh-7, an endogenously Lenvatinib-sensitive cell line, and the other from PLC, the most Lenvatinib-insensitive cell line. Following the selection of resistant clones over several months, we noticed that resistant Huh-7 cells (rHuh-7) were more than 20 times more resistant than parental Huh-7 (pHuh-7; IC50; 2.0 μM vs. 48.7 μM). Similarly, resistant PLC cell lines (rPLC) were more than two times more resistant than the parental PLC cells (pPLC; IC50; 44.3 μM vs. >100 μM; Figure 2A,B). Using these two acquired Lenvatinib-resistant cell lines, we performed a series of experiments to examine whether Curcumin might help sensitize these cells to Lenvatinib.

### 3.3. Curcumin Treatment Overcomes Acquired Lenvatinib Resistance via Inhibition of Proliferation, Invasion, and Colony Formation in HCC Cells

Firstly, we examined the antiproliferative effect of Curcumin and Lenvatinib combination in the newly established Lenvatinib-resistant HCC cell lines (Figure 2C). In both cell lines, co-administration of Curcumin exhibited the most potent anti-proliferative effects. The IC50 concentration of the combination in rHuh-7 cells was Curcumin: 3.6 μg/mL and Lenvatinib: 1.8 μM, and in rPLC cells, these concentrations were: Curcumin: 4.1 μg/mL and Lenvatinib: 2.0 μM (Figure 2C). At these concentrations, the proliferation inhibition by Curcumin treatment percentages were less than 25% in both cells (rHuh-7, 24.7%; rPLC, 22.1%; Figure 2C). Accordingly, the CI value of this combination at the dose of IC50 was 0.67 in rHuh-7 cells and 0.49 in the rPLC cell line (Figure 2D). These results indicated that the combination of Curcumin and Lenvatinib could help overcome the acquired resistance of Lenvatinib in HCC cells.

**Figure 3:**
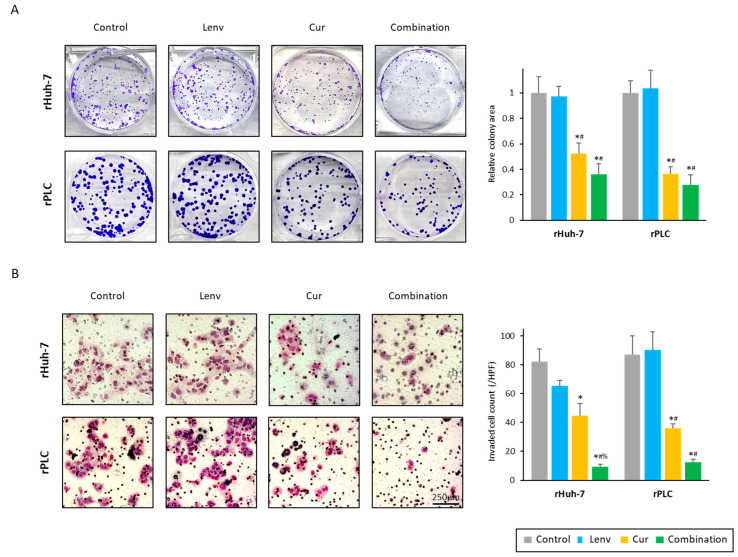
Curcumin reversed Lenvatinib resistance by inhibiting colony formation and invasion in HCC cell lines. (**A**) Colony formation assays for Lenvatinib-resistant cells following treatment (*: *p* < 0.05 vs. control, #: *p* < 0.05 vs. Lenvatinib). (**B**) Invasion assays following treatment in Lenvatinib-resistant cells. Scale bar = 250 μM. The number of invaded cells were randomly counted at three microscopic fields per membrane (*: *p* < 0.05 vs. control, #: *p* < 0.05 vs. Lenvatinib, %: *p* < 0.05 vs. Curcumin). Images show representative fields on the membrane (magnification 400×). The data indicate mean (column) ± SD values. SD, standard deviation.

Secondly, we examined the effects of this combination on the malignant potential of HCC cells. Colony formation ability was significantly reduced by Curcumin treatment in both rHuh-7 (relative colony area; control vs. Curcumin, 1.00 vs. 0.52, *p* < 0.01) and rPLC (relative colony area; control vs. Curcumin, 1.00 vs. 0.36, *p* < 0.01) compared to control and Lenvatinib treatment (Figure 3A). In addition, the combination treatment with both Curcumin and Lenvatinib exhibited a further reduction in colony formation by 64.9% in rHuh-7 cells and by 72.4% in the rPLC cell lines, compared to the untreated controls (*p* < 0.01; Figure 3A).

Thirdly, we evaluated the invasive ability of cancer cells using the trans-well invasion assays, where Curcumin treatment reduced tumor invasion by 54.5% in the rHuh-7 cell line and by 41.3% in the rPLC cells (Figure 3B). Moreover, the combined treatment with both compounds dramatically reduced the number of invaded cells in both rHuh-7 (control vs. combination, *p* < 0.01; Lenvatinib vs. combination, *p* < 0.01; Curcumin vs. combination, *p* < 0.01) and rPLC cells (control vs. combination, *p* < 0.01; Lenvatinib vs. combination, *p* < 0.01; Curcumin vs. combination, *p* < 0.12) (Figure 3B). In line with two resistant cell lines, Curcumin and combination treatment also weakened the malignant potential of SNU449 and parental PLC cells, the two endogenously Lenvatinib-insensitive cell lines (Figure S3).

### 3.4. Curcumin Treatment in Lenvatinib-Resistant Cells Led to Increased Accumulation of Reactive Oxygen Species and Induction of Apoptosis in HCC Cells

**Figure 4:**
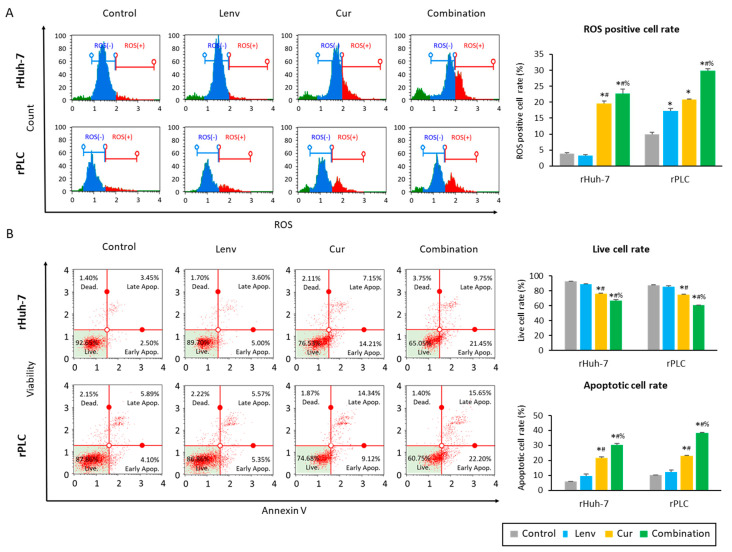
Co-administration of Curcumin with Lenvatinib induced cellular apoptosis via intracellular accumulation of ROS. (**A**) Representative images of oxidase stress assay following treatment in Lenvatinib-resistant cells (*: *p* < 0.05 vs. control, #: *p* < 0.05 vs. Lenvatinib, %: *p* < 0.05 vs. Curcumin). (**B**) Representative images of annexin-V assays. Graphs indicate the percentages of live and apoptotic cells (*: *p* < 0.05 vs. control, #: *p* < 0.05 vs. Lenvatinib, %: *p* < 0.05 vs. Curcumin). The data indicate mean (column) ± SD values. ROS, reactive oxygen species; SD, standard deviation.

Next, we examined the effect of Curcumin and Lenvatinib on the intracellular accumulation of reactive oxygen species (ROS) and apoptosis in HCC cells. The baseline percentages for the ROS-positive cell population were 3.9% (rHuh-7) and 9.9% (rPLC; Figure 4A). In both cell lines, intracellular ROS levels increased more than two times by Curcumin treatment alone (rHuh-7, 19.5%; rPLC, 20.9%) and by more than three times in following the combination treatment (rHuh-7, 22.7%; rPLC: 29.8%; Figure 4A).

In line with these findings, the cellular apoptosis rates were also increased by Curcumin and the combination treatments (Figure 4B). The baseline live cell rates were 92.6% in rHuh-7 and 87.6% in rPLC cells, and not surprisingly, Lenvatinib treatment had no impact on the live cell rates in resistant cell lines (rHuh-7; 88.5%, rPLC; 85.5%, Figure 4B). However, Curcumin treatment alone and in combination with Lenvatinib significantly reduced live cell rates in both rHuh-7 cells (Curcumin, 75.9%; combination, 66.6%) and rPLC cell lines (Curcumin, 75.0%; combination, 60.4%) (Figure 4B). Moreover, more than 30% of cells underwent apoptosis following Curcumin and Lenvatinib combination treatment, with significantly higher induction of apoptosis in rHuh-7 cells (apoptotic cell rate: control vs. Lenvatinib vs. Curcumin vs. combination, 6.0% vs. 9.7% vs. 21.8% vs. 30.2%; control vs. combination, *p* < 0.01; Lenvatinib vs. combination, *p* < 0.01; Curcumin vs. combination, *p* < 0.01) and rPLC cells (apoptotic cell rate: control vs. Lenvatinib vs. Curcumin vs. combination, 10.1% vs. 12.2% vs. 23.1% vs. 38.2%; control vs. combination, *p* < 0.01; Lenvatinib vs. combination, *p* < 0.01; Curcumin vs. combination, *p* < 0.01; Figure 4B). From these results, we could glean that co-administration of Curcumin and Lenvatinib led to the induction of apoptosis via increased accumulation of ROS in Lenvatinib-resistant cells, eventually supporting the enhanced anti-tumor efficacy of this combination treatment in HCC cells.

### 3.5. Acquired Lenvatinib Resistance Is Mediated by Activation of the EGFR Pathway in HCC Cells

**Figure 5:**
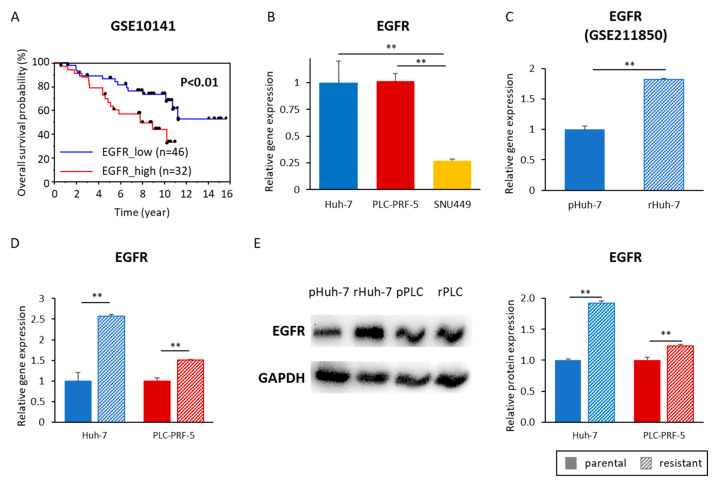
EGFR activation was associated with the poor prognosis in HCC patients and acquired resistance. (**A**) Survival curves of high and low expression of EGFR among patients with HCC. (**B**) EGFR gene expression in Huh-7, PLC-PRF, and SNU449 cell lines (**: *p* < 0.01). (**C**) EGFR gene expression in parental and resistant Huh-7 (GSE211850) cell lines (**: *p* < 0.01). (**D**) EGFR gene expression in parental and resistant Huh-7 and PLC-PRF-5 (GSE211850) cell lines (**: *p* < 0.01). (**E**) Western blotting of EGFR expression in parental and resistant Huh-7 and PLC-PRF-5 cell lines. GAPDH was used as an internal control protein. (**: *p* < 0.01). The data indicate mean (column) ± SD values. SD, standard deviation.

Recent studies have suggested that EGFR activation might be associated with Lenvatinib resistance in HCC [21,22,23]. Survival analysis of HCC patients (GSE10141) revealed that EGFR activation in cancer tissues was an independent prognostic factor in patients with HCC (*p* < 0.01, Figure 5A). We next investigated whether EGFR activation is also associated with the acquisition of Lenvatinib resistance in HCC cells. For these experiments, we first compared the gene expression of the EGFR gene in Huh-7, SNU449, and PLC cell lines. We did not observe any change in EGFR expression between Huh-7 (Lenvatinib-sensitive) and the PLC cells (Lenvatinib-insensitive; Figure 5B). However, SNU449 cells (another Lenvatinib-insensitive cell line) demonstrated a significantly lower expression of EGFR than Huh-7 (*p* < 0.01) and PLC (*p* < 0.01) cells, suggesting that EGFR expression might not be associated with the endogenous sensitivity to Lenvatinib (Figure 5B). More importantly, when we compared the EGFR gene expression between parental Huh-7 (pHuh-7) and resistant Huh-7 cells in a publicly available dataset (GSE211850), EGFR expression was significantly upregulated in rHuh-7 cells (pHuh-7 vs. rHuh-7, 1.00 vs. 1.82, *p* < 0.01; Figure 5C). We were subsequently able to successfully validate this observation for EGFR upregulation in our newly established Lenvatinib-resistant cell lines (Huh-7: parental vs. resistant 1.00 vs. 2.57, *p* < 0.01; PLC: parental vs. resistant, 1.00 vs. 1.51, *p* < 0.01; Figure 5D). We further confirmed the EGFR protein expression and revealed that EGFR protein expression in resistant cell lines was 1.92 times higher in rHuh-7 (*p* < 0.01) and 1.23 times higher in rPLC (*p* < 0.01) compared to the parental cell lines (Figure 5E). Collectively, these results indicated that EGFR activation is an important prognostic factor of HCC and relates to acquired Lenvatinib resistance, highlighting that EGFR and its downstream pathway might be a promising therapeutic target of Lenvatinib resistance in this malignancy.

### 3.6. Curcumin-Mediated Sensitization to Lenvatinib Is Orchestrated via Suppression of EGFR and Its Downstream PI3K-AKT Pathway

To identify specific EGFR downstream pathways associated with Lenvatinib resistance, we performed genomewide expression profiling analysis between parental and resistant Huh-7 cells (GSE211850). There were 2522 significantly upregulated genes (log2 Fold change > 1, *p* < 0.05) in rHuh-7 compared to parental cells. The KEGG pathway analysis for these upregulated genes is depicted in Table S3. Interestingly, the EGFR tyrosine kinase inhibitor resistance pathway (gene count; 17, fold enrichment; 1.6) was identified among the top 50 enrichment pathways. Moreover, the PI3K-Akt signaling pathway, a major downstream pathway of EGFR, was identified as the fourth highest-ranked pathway (gene count; 59, fold enrichment; 1.3, Table S3). These results suggested that the PI3K-AKT pathway is associated with Lenvatinib resistance in HCC cells, and led us to focus on the EGFR-PI3K-AKT pathway and examine the effects of Curcumin for its ability to suppress this signaling pathway.

**Figure 6:**
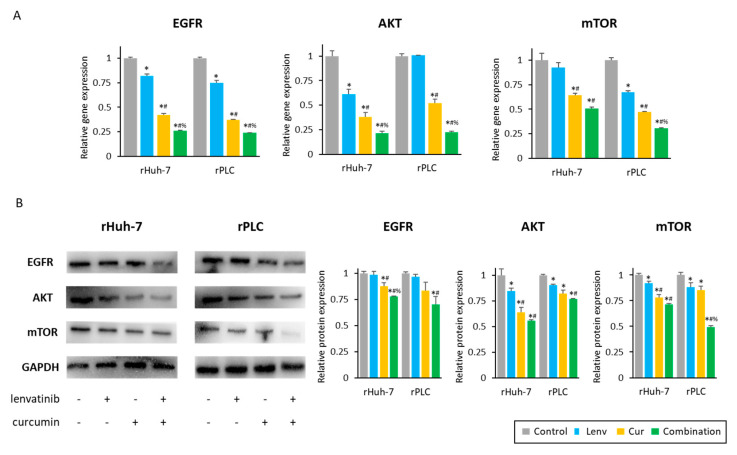
A combination of Curcumin and Lenvatinib downregulated the activity of EGFR and its downstream signaling pathway in Lenvatinib-resistant cells. (**A**) Gene expression changes of EGFR, AKT, and mTOR genes (*: *p* < 0.05 vs. control, #: *p* < 0.05 vs. Lenvatinib, %: *p* < 0.05 vs. Curcumin). (**B**) Western blotting of EGFR, AKT, and mTOR expression in Huh-7 and PLC-PRF-5 cell lines. GAPDH was used as an internal control protein (*: *p* < 0.05 vs. control, #: *p* < 0.05 vs. Lenvatinib, %: *p* < 0.05 vs. Curcumin). The data indicate mean (column) ± SD values. SD, standard deviation.

Curcumin significantly suppressed EGFR gene expression in rHuh7 (control vs. Curcumin, 1.00 vs. 0.42, *p* < 0.01) and rPLC cells (control vs. Curcumin, 1.00 vs. 0.37, *p* < 0.01; Figure 6A). Furthermore, co-administration of Curcumin with Lenvatinib further reduced EGFR expression in both rHuh-7 (Curcumin vs. combination, 0.42 vs. 0.26, *p* < 0.01) and rPLC cell lines (Curcumin vs. combination, 0.37 vs. 0.24, *p* < 0.01), compared to Curcumin treatment alone (Figure 6A). Likewise, the EGFR protein expression also was significantly decreased by Curcumin (rHuh-7, 0.88; rPLC; 0.84) and the combination treatment (rHuh-7, 0.78; rPLC, 0.70; Figure 6B) compared to a non-treated group, suggesting that Curcumin-mediated suppression of EGFR might be one of the mechanisms for sensitizing Lenvatinib-resistant HCC cells. Although EGFR gene expression was decreased by Lenvatinib monotherapy (rHuh-7, 0.82; rPLC; 0.75; Figure 6A), its protein expression was not changed (rHuh-7, 0.99; rPLC; 0.97; Figure 6B), suggesting Lenvatinib only has a weak effect on modulating the expression of EGFR.

We next examined the expression of AKT and mTOR genes, which are critical modulators of the PI3K-AKT pathway. Curcumin treatment resulted in significant down-regulation of AKT and mTOR gene expression compared to untreated controls and Lenvatinib treatment (Figure 6A). In addition, the combination treatment with Curcumin and Lenvatinib further decreased AKT and mTOR gene expression (AKT: 0.21 in rHuh-7, 0.22 in rPLC; mTOR: 0.51 in rHuh-7, 0.31 in rPLC, Figure 6A). In line with the gene expression results, the protein expression of AKT and mTOR were similarly suppressed by both curcumin and the combination treatment in both rHuh-7 (AKT: control vs. Curcumin vs. combination, 1.00 vs. 0.64 vs. 0.56; mTOR: control vs. Curcumin vs. combination, 1.00 vs. 0.78 vs. 0.71) and rPLC cell lines (AKT: control vs. Curcumin vs. combination, 1.00 vs. 0.82 vs. 0.77; mTOR: control vs. Curcumin vs. combination, 1.00 vs. 0.85 vs. 0.49; Figure 6B). These results supported our hypothesis that Curcumin overcomes Lenvatinib resistance in HCC via suppression of the EGFR-PI3K-AKT pathway.

### 3.7. Curcumin Treatment Suppresses Cancer Stemness in Lenvatinib-Resistant HCC Cells

**Figure 7:**
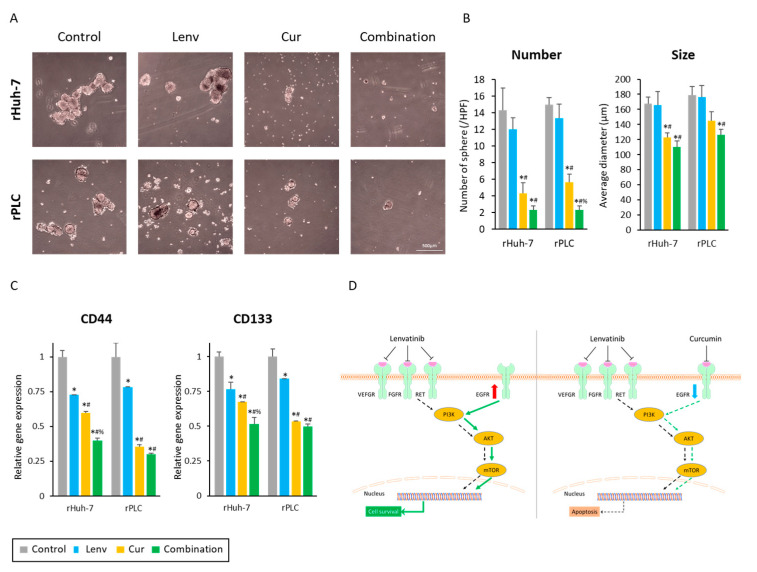
Co-administration of Lenvatinib with Curcumin diminished the spheroid-forming ability via suppression of cancer stemness markers. (**A**) Representative images of cancer spheroids from Lenvatinib-resistant cells following treatment (magnification 100×). Scale bar = 500 μM. (**B**) Number and size of spheroids following treatment (*: *p* < 0.05 vs. control, #: *p* < 0.05 vs. Lenvatinib, %: *p* < 0.05 vs. Curcumin). (**C**) Gene expression changes in CD44 and CD133 genes (*: *p* < 0.05 vs. control, #: *p* < 0.05 vs. Lenvatinib, %: *p* < 0.05 vs. Curcumin). (**D**) A schematic illustration of the Curcumin and Lenvatinib combination treatment overcoming Lenvatinib resistance via suppression of EGFR and its downstream signaling. The data indicate mean (column) ± SD values. SD, standard deviation.

To further examine the anti-tumorigenic effects of the Curcumin and Lenvatinib combination treatment in cancer stem cells (CSC), we developed cancer spheroids from two Lenvatinib-resistant cells. Following the completion of treatments, the number and size of spheroids were evaluated. The Curcumin treatment and Lenvatinib significantly reduced spheroids’ number and size (Figure 7A). The number of spheroids decreased from 14.3 (control) to 4.3 (Curcumin, *p* < 0.01) and 2.3 (combination, *p* < 0.01) in rHuh-7 cells, and from 15.0 (control) to 5.7 (Curcumin, *p* < 0.01) and 2.3 (combination, *p* < 0.01) in rPLC cell line (Figure 7B, left panel). Similarly, the mean spheroid size was also decreased by Curcumin and the combination treatment in rHuh-7 cells (control vs. Curcumin vs. combination, 167.7 μM vs. 122.9 μM vs. 110.6 μM; control vs. Curcumin, *p* = 0.02, control vs. Combination, *p* < 0.01) and rPLC cells (control vs. Curcumin vs. combination, 178.9 μM vs. 144.8 μM vs. 126.6 μM; control vs. Curcumin, *p* = 0.08, control vs. Combination, *p* = 0.01; Figure 7B, right panel).

We also evaluated the expression of CD44 and CD133, which are the cancer stemness markers in many cancers, including HCC [40]. Interestingly, although there were no significant changes in the number and size of spheroids in untreated controls and Lenvatinib-treated cells (Figure 7A,B), both cancer stemness markers were about 25% downregulated by Lenvatinib treatment in both lines (Figure 7C). More importantly, Curcumin and the combination treatment significantly reduced the expression of both markers compared to the untreated controls and Lenvatinib-treated cells (Figure 7C). Although there were no significant differences in the expression of CD44 and CD133 between curcumin-treated and the combination treatment groups, co-administration of Curcumin and Lenvatinib exhibited the most potent inhibition of gene expression for both CSC markers (CD44: 0.40 in rHuh-7, 0.30 in rPLC; CD133: 0.52 in rHuh7, 0.50 in rPLC, Figure 7C). These findings from cancer spheroid assays strengthened our results for suppressing a CSC phenotype as a mechanism of Curcumin-mediated reversal of Lenvatinib resistance in HCC.

## 4. Discussion

Hepatocellular carcinoma (HCC) is the most common primary liver malignancy and is known to be a highly malignant tumor type due to its poor prognosis [1]. Unresectable advanced HCC patients are treated with systemic therapies, and currently, Sorafenib [2], Lenvatinib [3], and Atezolizumab plus Bevacizumab [4] are approved as first-line treatments. Recent evidence suggests that Lenvatinib is more effective than Atezolizumab plus Bevacizumab for HCC patients with non-viral etiology, which is currently rising [9,10]. However, the overall response rates of Lenvatinib are ~24% [19], and patients who initially respond to this treatment eventually acquire resistance to this therapeutic modality. Although the molecular trigger for Lenvatinib resistance remains unclear, recent studies have suggested that EGFR activation is frequently observed in Lenvatinib-resistant cell lines [21,22]. Therefore, the combination of Lenvatinib and an EGFR inhibitor is considered to have clinical application potential in HCC. However, there are still concerns about the toxicity and high costs associated with such combination therapies.

Curcumin is a naturally-occurring botanical derived from turmeric and is one of the most widely studied, safe and inexpensive adjunctive treatment approaches used in various human cancers [25,29,30,31]. Curcumin exhibits its anti-cancer efficacy by modulating many important cancer-related processes, including inflammation, cell cycle, and apoptosis [33,35,36]. More importantly, previous evidence also indicates that Curcumin acts as a tyrosine kinase inhibitor [36,37]. Therefore, in the present study, we hypothesized that Curcumin might enhance the anti-tumor effects of Lenvatinib as a tyrosine kinase inhibitor and that the anti-EGFR potential of Curcumin could help overcome Lenvatinib resistance in HCC.

According to our hypothesis, Curcumin enhanced the anti-tumor effects of Lenvatinib in both endogenously sensitive and relatively insensitive HCC cell lines by inhibiting cell proliferation, invasion, and colony formation ability. Moreover, we noted that Curcumin could reverse acquired resistance to Lenvatinib by suppressing the EGFR-PI3K-AKT signaling cascade (Figure 7D). In this context, co-administration of Curcumin with Lenvatinib suppressed cellular proliferation, invasion, and colony formation, along with induction of cellular apoptosis via intracellular accumulation of ROS in both parental and Lenvatinib-resistant cells. To better understand this mechanistic observation, we established two Lenvatinib-resistant cell lines, one from Huh-7, which is an endogenously Lenvatinib-sensitive cell line, and the other from PLC, which represents the most Lenvatinib-insensitive cell line. These newly established Lenvatinib-resistant cell lines showed EGFR activation, which supports the suggestions in some previous reports [21,22]. Genomewide analysis of Lenvatinib-resistant cells identified the PI3K-AKT pathway, a major downstream signaling event of EGFR, as a therapeutic target for Lenvatinib-resistance in HCC. We successfully demonstrated that Curcumin could suppress this signaling pathway in Lenvatinib-resistant cells. Moreover, co-administration of Curcumin with Lenvatinib significantly diminished the spheroid-forming ability of these resistant cancer cells via suppression of cancer stemness markers, reinforcing our findings of this combination in overcoming Lenvatinib resistance.

Our findings can potentially provide a new adjunctive therapeutic strategy in HCC. However, there were a few potential limitations to our study that we would like to acknowledge. First, we revealed that EGFR inhibition by Curcumin could overcome the acquired resistance of Lenvatinib using genomewide analysis and following experiments. However, we have not investigated how Curcumin could enhance the anti-tumor effect of Lenvatinib on endogenously insensitive cells. Secondly, we examined the effects of combination treatment in cancer cells and cancer spheroids but not in pre-clinical animal models of HCC. While this might provide additional insight, unfortunately, no robust animal models capture human HCC. In addition, although Curcumin is a safe compound, we did not directly evaluate any potential toxicity when combined with Lenvatinib. In the future, validation studies for the effect and toxicity of this combination in such animal experimental models or clinical studies might shed additional insight, given the safety profile of Curcumin with other chemotherapies and targeted drugs reported to date, the likelihood of any adverse combinatorial effects are highly unlikely.

## 5. Conclusions

In conclusion, we provide the first evidence that Curcumin could reverse Lenvatinib resistance in HCC cells via suppression of EGFR and its downstream targets, which can offer a novel adjunctive, safe and efficacious therapeutic strategy in HCC.

## Data Availability

Data is contained within the article and Supplemental Materials.

## Supplementary Materials

The following supporting information can be downloaded at: https://www.mdpi.com/article/10.3390/cells12040612/s1, Figure S1: Cell viability assay for a lower concentration of Lenvatinib in Huh-7 cells; Figure S2: Isobologram analysis of Curcumin and Lenvatinib combination in resistant Huh-7 and PLC-PRF-5 cell lines; Figure S3: Curcumin enhanced the anti-tumor effects of Lenvatinib by inhibiting colony formation and invasion; Figure S4: Original Western Blots; Table S1: the list of primer sequences; Table S2: the list of antibodies; Table S3: KEGG pathway analysis.
