# Supplementary material for: Curcumin-Mediated Resistance to Lenvatinib via EGFR Signaling Pathway in Hepatocellular Carcinoma
Source: Cells. 2023 Feb 14;12(4):612. doi: 10.3390/cells12040612 (PMC9954241; doi:10.3390/cells12040612)
Supplement: Supplementary file 1 [file cells-12-00612-s001.zip › cells-2171263-supplementary.pdf]

Table S1. the list of primer sequences

Gene symbol	Forward sequence	Reverse sequence
EGFR	TTGCCGCAAAGTGTGTAACG	GTCACCCCTAAATGCCACCG
AKT	GCTGGACGATAGCTTGGA	GATGACAGATAGCTGGTG
mTOR	GCAGATTTGCCAACTATCTTCGG	CAGCGGTAAAAGTGTCCCCTG
CD44	CAATAGCACCTTGCCCACAAT	AATCACCACGTGCCCTTCTATGG
CD133	CAGAGTACAACGCCAAACCA	AAATCACGATGAGGGTCAGC
GAPDH	TGAACGGGAAGCTCACTG	TCCACCACCCTGTTGCTGTA

Table S2. the list of antibodies

Target	ID	Company
EGFR	#4267	Cell Signaling Technology, MA, USA
AKT	#4691	Cell Signaling Technology, MA, USA
mTOR	#4517	Cell Signaling Technology, MA, USA
GAPDH	10494-1-AP	Proteintech Group Inc, IL, USA
Anti-rabbit IgG	#7074	Cell Signaling Technology, MA, USA
Anti-mouse IgG	#7076	Cell Signaling Technology, MA, USA

Table S3. KEGG pathway analysis

KEGG pathway	Gene count	Fold enrichment	P-Value
Metabolic pathways	272	1.3	1.60E-08
Pathways in cancer	94	1.3	1.80E-03
Human papillomavirus infection	64	1.5	1.30E-03
**PI3K-Akt signaling pathway**	59	1.3	4.20E-02
MAPK signaling pathway	57	1.5	2.30E-03
Lysosome	50	2.9	2.00E-12
Salmonella infection	46	1.4	1.50E-02
Endocytosis	45	1.4	2.60E-02
Rap1 signaling pathway	44	1.6	1.80E-03
Human cytomegalovirus infection	41	1.4	2.70E-02
Shigellosis	41	1.3	9.20E-02
Protein processing in the endoplasmic reticulum	40	1.8	3.20E-04
Chemical carcinogenesis - reactive oxygen species	40	1.4	3.60E-02
Autophagy - animal	38	2	2.00E-05
Pathogenic Escherichia coli infection	38	1.5	1.50E-02
Human T-cell leukemia virus 1 infection	38	1.3	7.30E-02
Axon guidance	36	1.5	1.20E-02
Kaposi sarcoma-associated herpesvirus infection	36	1.4	3.00E-02
Focal adhesion	36	1.4	4.80E-02
Wnt signaling pathway	33	1.5	2.10E-02
Hippo signaling pathway	31	1.5	2.10E-02
Cell adhesion molecules	30	1.5	3.50E-02
Alcoholic liver disease	29	1.6	1.70E-02
FoxO signaling pathway	28	1.6	1.10E-02
Cellular senescence	28	1.4	7.90E-02
Ubiquitin mediated proteolysis	27	1.4	4.80E-02

Phospholipase D signaling pathway	27	1.4	7.30E-02
Gastric cancer	27	1.4	7.80E-02
Apoptosis	25	1.4	7.90E-02
Fluid shear stress and atherosclerosis	25	1.4	9.60E-02
Pancreatic cancer	24	2.4	7.10E-05
Neurotrophin signaling pathway	24	1.5	3.50E-02
Insulin resistance	23	1.6	2.30E-02
Th17 cell differentiation	23	1.6	2.30E-02
Growth hormone synthesis, secretion, and action	23	1.5	6.40E-02
AGE-RAGE signaling pathway in diabetic complications	22	1.7	1.90E-02
Sphingolipid signaling pathway	22	1.4	9.50E-02
Adherens junction	21	2.2	5.90E-04
Small cell lung cancer	20	1.7	2.90E-02
Choline metabolism in cancer	20	1.6	5.10E-02
Parathyroid hormone synthesis, secretion, and action	20	1.4	9.70E-02
Peroxisome	19	1.8	1.80E-02
Colorectal cancer	19	1.7	2.90E-02
Bile secretion	19	1.6	3.90E-02
Phosphatidylinositol signaling system	19	1.5	8.00E-02
Epithelial cell signaling in Helicobacter pylori infection	18	2	7.90E-03
GnRH signaling pathway	18	1.5	9.70E-02
Notch signaling pathway	17	2.2	3.10E-03
Mitophagy - animal	17	1.8	2.30E-02
**EGFR tyrosine kinase inhibitor resistance**	17	1.6	4.90E-02

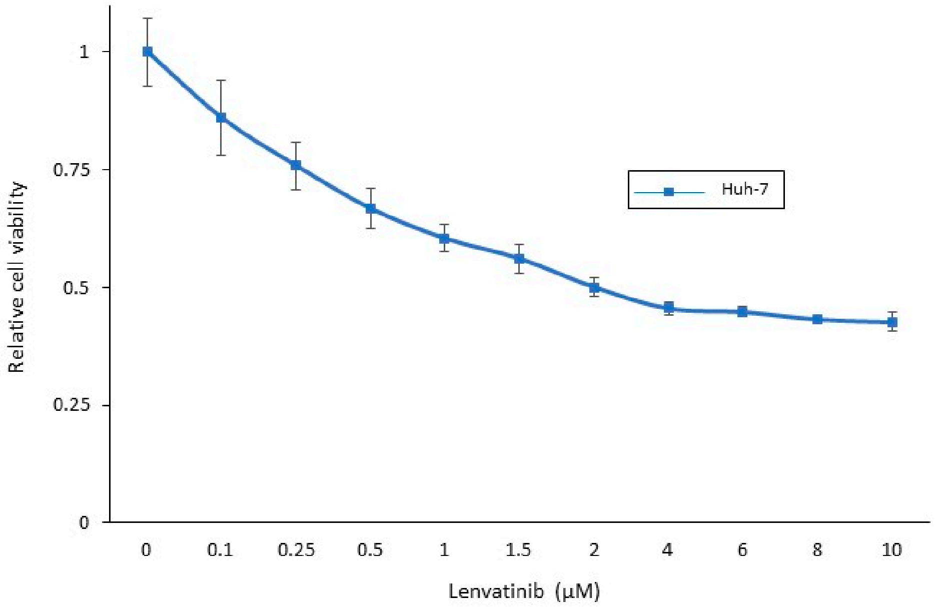

Figure S1. Cell viability assay for a lower concentration of Lenvatinib in Huh-7 cells.

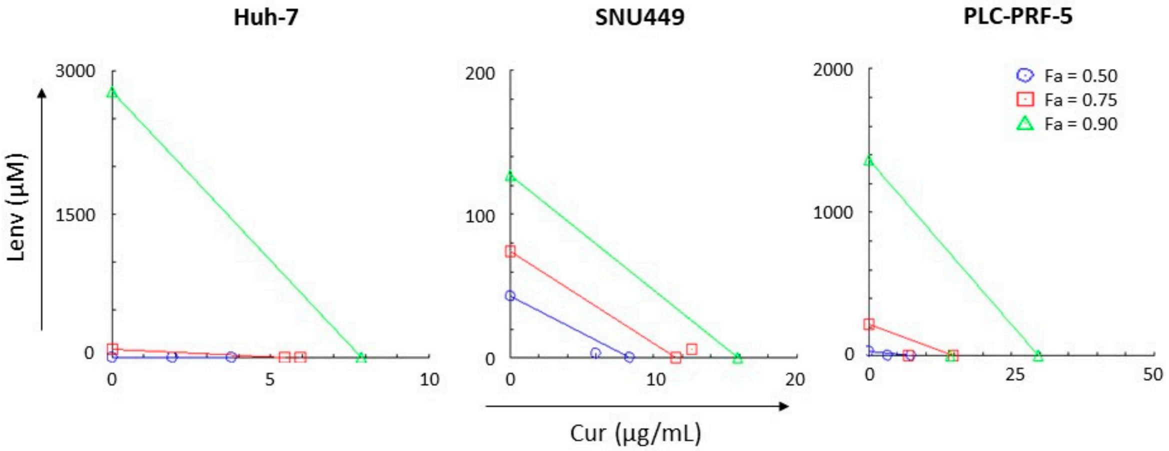

Figure S2. Isobologram analysis of Curcumin and Lenvatinib combination in resistant Huh-7 and PLC-PRF-5 cell lines.

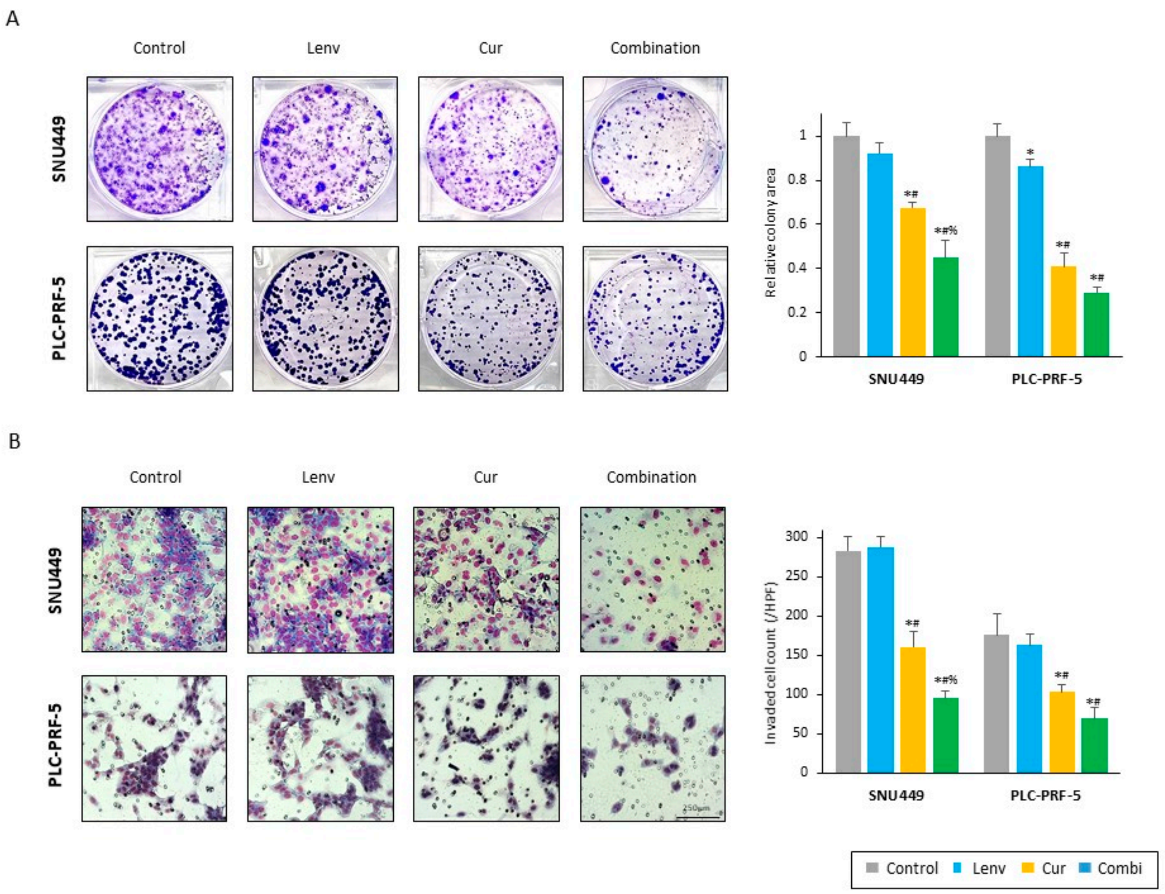

Figure S3. Curcumin enhanced the anti-tumor effects of Lenvatinib by inhibiting colony formation and invasion. A) Colony formation assays of SNU449 and PLC-PRF-5 following treatment (*: $p<0.05$ vs. control, #: $p < 0.05$ vs. Lenvatinib, %: $p < 0.05$ vs. Curcumin). B) Invasion assays following treatment in SNU449 and PLC-PRF-5. Scale bar = 250 μm. The number of invaded cells was randomly counted at three fields per membrane (*: $p<0.05$ vs. control, #: $p < 0.05$ vs. Lenvatinib, %: $p < 0.05$ vs. Curcumin). Images show representative fields on the membrane (magnification x400). The data indicate mean (column) ± SD values. SD, standard deviation.

Figure 5

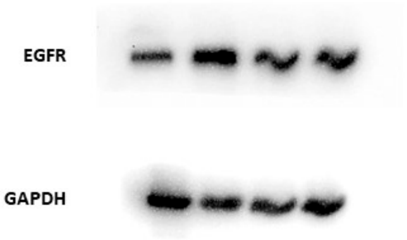

Figure 6

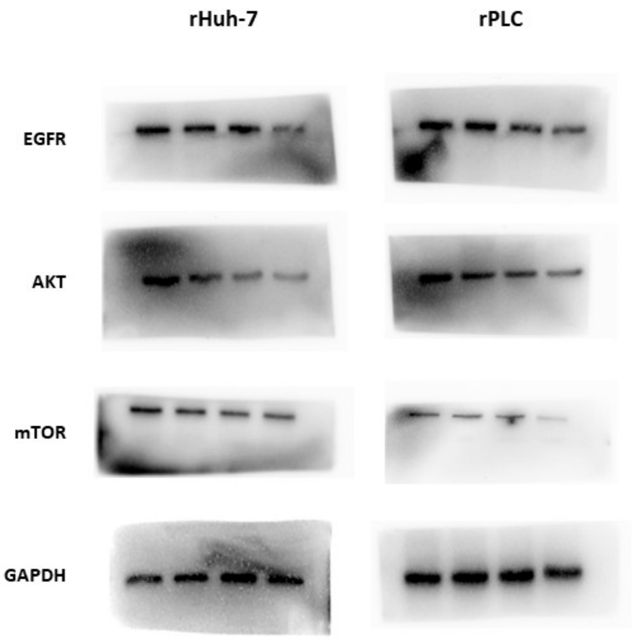

Figure S4. Original Western Blots
